# Galectin-3 in Prostate Cancer Stem-Like Cells Is Immunosuppressive and Drives Early Metastasis

**DOI:** 10.3389/fimmu.2020.01820

**Published:** 2020-09-10

**Authors:** Sara Caputo, Matteo Grioni, Chiara S. Brambillasca, Antonella Monno, Arianna Brevi, Massimo Freschi, Ignazio S. Piras, Angela R. Elia, Valentina Pieri, Tania Baccega, Angelo Lombardo, Rossella Galli, Alberto Briganti, Claudio Doglioni, Elena Jachetti, Matteo Bellone

**Affiliations:** ^1^Cellular Immunology Unit, Division of Immunology, Transplantation and Infectious Diseases, IRCCS San Raffaele Scientific Institute, Milan, Italy; ^2^NET-IMPACT, IRCCS San Raffaele Scientific Institute, Milan, Italy; ^3^Vita-Salute San Raffaele University, Milan, Italy; ^4^Innate Immunity and Tissue Remodeling Unit, Division of Immunology, Transplantation and Infectious Diseases, IRCCS San Raffaele Scientific Institute, Milan, Italy; ^5^Unit of Pathology, IRCCS San Raffaele Scientific Institute, Milan, Italy; ^6^Neurogenomics Division, Center for Rare Childhood Disorders (C4RCD), Translational Genomics Research Institute, Phoenix, AZ, United States; ^7^Neural Stem Cell Biology Unit, Division of Neuroscience, San Raffaele Scientific Institute, Milan, Italy; ^8^San Raffaele Telethon Institute for Gene Therapy (SR-Tiget), IRCCS San Raffaele Scientific Institute, Milan, Italy; ^9^Unit of Urology and URI, Division of Oncology, IRCCS Ospedale San Raffaele, Milan, Italy

**Keywords:** prostate cancer, cancer stem cell, Galectin-3, immunosuppression, immune surveillance, prostate intraepithelial neoplasia, metastasis, T lymphocytes

## Abstract

Galectin-3 (Gal-3) is an extracellular matrix glycan-binding protein with several immunosuppressive and pro-tumor functions. The role of Galectin-3 in cancer stem-like cells (CSCs) is poorly investigated. Here, we show that prostate CSCs also colonizing prostate-draining lymph nodes of transgenic adenocarcinoma of the mouse prostate (TRAMP) mice overexpress Gal-3. Gal-3 contributes to prostate CSC-mediated immune suppression because either Gal-3 silencing in CSCs, or co-culture of CSCs and T cells in the presence of the Gal-3 inhibitor N-Acetyl-D-lactosamine rescued T cell proliferation. N-Acetyl-D-lactosamine also rescued the proliferation of T cells in prostate-draining lymph nodes of TRAMP mice affected by prostate intraepithelial neoplasia. Additionally, Gal-3 impacted prostate CSC tumorigenic and metastatic potential *in vivo*, as Gal-3 silencing in prostate CSCs reduced both primary tumor growth and secondary invasion. Gal-3 was also found expressed in more differentiated prostate cancer cells, but with different intracellular distribution as compared to CSCs, which suggests different functions of Gal-3 in the two cell populations. In fact, the prevalent nuclear and cytoplasmic distribution of Gal-3 in prostate CSCs made them less susceptible to apoptosis, when compared to more differentiated prostate cancer cells, in which Gal-3 was predominantly intra-cytoplasmic. Finally, we found Gal-3 expressed in human and mouse prostate intraepithelial neoplasia lesions and in metastatic lymph nodes. All together, these findings identify Gal-3 as a key molecule and a potential therapeutic target already in the early phases of prostate cancer progression and metastasis.

## Introduction

Cancer is a multifactorial disease in which genetic and environmental factors concomitantly and progressively lead to neoplastic transformation and tumor development (1). According to the hierarchical model of cancer evolution, cancer stem-like cells (CSCs) represent the subpopulation of cancer cells within the tumor bulk that are endowed with tumorigenic potential, thus driving tumor growth and metastasis (2, 3). Nevertheless, cancer cells are not solitary entities, as they are embedded within the tumor microenvironment, which is composed of several players among which stromal cells (4), immune cells (5), and non-cellular components such as collagen fibers and glycosylated molecules, constituting the extracellular matrix (6, 7). A constant crosstalk between cancer cells and the tumor microenvironment ensures tumor development and progression.

Galectin-3 (Gal-3) is an extracellular matrix glycan-binding protein whose function spans several biological processes, including immune modulation, chemoattraction, cell adhesion, activation, differentiation, and apoptosis (8). Gal-3 is involved in several pathological events, and the function of Gal-3 depends on its location within the cell. When expressed on the plasma membrane or secreted, Gal-3 interacts with glycoconjugates, i.e., carbohydrate structures linked to lipids, proteins and peptides, thus mediating cell-cell, and cell-matrix interactions. In the cytoplasm, Gal-3 participates to the regulation of cell growth, cell cycle progression, and may inhibit apoptosis. Conversely, when localized into the nucleus, Gal-3 is pro-apoptotic. Given its pleiotropic effects, Gal-3 has been referred to as: “the guardian of the tumor microenvironment” (9).

Gal-3 is variably expressed in tumors, where it favors malignant transformation, invasion, and metastasis, and can also exert immunosuppressive functions (10, 11).

Gal-3 has been extensively investigated in prostate cancer (12). The healthy human prostate epithelium shows moderate immunostaining for Gal-3 that is localized both in the nucleus and the cytoplasm (13–16). In prostate intraepithelial neoplasia (PIN) lesions, Gal-3 expression is mainly cytoplasmic, more heterogeneous and more intense than in non-tumoral epithelium, but with a lower percentage of positive cells (14). Gal-3 expression is further reduced in prostate adenocarcinoma (13, 15), likely due to promoter methylation (17). Indeed, Gal-3 appears to be cleaved upon disease progression (18). However, in one report of 145 prostate cancer patients subjected to radical prostatectomy, the intensity of Gal-3 expression in carcinoma cells significantly associated with prostate specific antigen (PSA) relapse in univariate analysis, and exclusive cytoplasmic localization of Gal-3 was an independent prognostic indicator of biochemical progression after radical prostatectomy (14). Thus, localization within some cancer cells rather then percentage of tumor cells expressing Gal-3 seems relevant in prostate cancer. Additionally, Gal-3 favors prostate cancer metastasis, and oral administration of modified citrus pectin reduced the number of lung metastases in rats (19). Gal-3 has also been suggested as complementary diagnostic marker to PSA blood test, as serum concentration of Gal-3 was found increased in metastatic prostate cancer patients when compared to healthy subjects (20). Intriguingly, Gal-3 has also been proposed as potential biomarker at early clinical stages of prostate cancer (21).

Although little is known about Gal-3 function in CSCs, Gal-3 expression has been reported in CSCs from ovarian, gastrointestinal, kidney, and lung tumors (22–26). CSCs have been implicated in the development and progression of primary lesions and metastases (27). How precociously CSCs invade sites of prospective clinical metastasis still needs to be defined. Two general paradigms explain the process of systemic cancer progression (28). The linear progression model establishes that tumor ontogeny fully occurs in the primary tumor, and identifies metastasis as a late event that follows the development of a large tumor bulk. Consequently, metastases and primary tumor share genetic similarities. Conversely, the parallel progression model posits that tumor cells leave the primary lesion before acquisition of full malignant phenotype, and migrate to secondary sites where they acquire additional genetic hits. Thus, great genetic and epigenetic disparities characterize primary tumor cells and metastasis founders. We have previously reported that CSCs obtained from mouse PIN lesions [hereafter named as TPIN-SCs; ref. (29)], and concomitantly, from histopathology negative prostate draining lymph nodes were phenotypically and functionally identical, thus suggesting a common origin (30), and demonstrating that lymph node invasion may already occur at the early stage of PIN in transgenic adenocarcinoma of the mouse prostate (TRAMP) mice (31). Thus, our results support the hypothesis that prostate cancer adheres to the parallel progression model of metastasis.

Because Gal-3 is a key molecule involved in several aspects of tumor progression and metastasis (10), and our previous data suggested that TPIN-SCs over-express the Gal-3 transcript (29, 30), here we further investigated the expression of Gal-3 in TPIN-SCs, and asked if Gal-3 expressed by prostate CSCs plays a relevant role in the neoplastic process. Our findings demonstrate that already at the stage of mouse PIN, Gal-3 has a pivotal role in balancing tumorigenic, metastatic, and immunosuppressive abilities in CSCs.

## Materials and Methods

### Mice

C57BL/6, C57BL/6-Tg(TcraTcrb)425Cbn/Crl, C57BL/6-Tg(TcraTcrb)1100Mjb/Crl (Charles River, Calco, Italy), and B6.129S7-Rag1tm1Mom/J mice (32) were housed in a pathogen-free animal facility. The latter two mouse strains were crossed to obtain RAG-1^−/−^ OT1 mice. Heterozygous TRAMP mice (31) were generated as described (33). NOD.Cg-PrkdcscidIl2rgtm1Wjl/SzJ mice are also known as NOD scid gamma (NSG; Charles River, Calco, Italy). Animals were treated in accordance with the European Community guidelines and with the approval of the Institutional Ethical Committee.

### Cell Culture

TPIN071122 and TNE070116 cells and the newly obtained TPIN1323 CSCs were cultured in NeuroCult™ NS-A Basal Medium (STEMCELL TECHNOLOGIES) supplemented with heparin, EGF, and bFGF according to the manufacturing instructions, as described previously (29). Murine splenocytes were cultured in T cell medium (TCM), composed by RPMI (Lonza), with 10% fetal bovine serum (FBS; Invitrogen, Milan, Italy), 2 mM L-glutamine, 150 U/ml streptomycin and 200 U/ml penicillin (Cambrex, Milan, Italy), 10 mM Hepes, 10 mM Sodium Pyruvate and 5 μM β-mercaptoetanol (Gibco-Invitrogen, Milan, Italy). TRAMP-C2 cells (34) were cultured in DMEM (Lonza), with 10% FBS (Invitrogen). Unless specified, all chemical reagents were from Sigma-Aldrich (St. Louis, MO, USA). Peptides were kindly provided by R. Longhi (CNR, Milan, Italy). Human Du145 (35) and PC-3 cells (36) were cultured in RPMI (Lonza), with 10% FBS (Invitrogen).

### Proliferation Assays

Splenocytes were labeled with the fluorescent dye carboxyfluorescein diacetate succinimidyl ester [CFSE, ref. (37)], and activated *in vitro* with anti-CD3 and anti-CD28 beads (Invitrogen) and IL-2 (R&D Systems, Minneapolis, MN, USA) according to the manufacturer instructions. When needed, irradiated (50 Gy) prostate CSC were added in co-culture at CSC:splenocyte ratio that corresponds to 1:10. When indicated, activated splenocytes were treated with 5 mM N-Acetyl-D-lactosamine (LacNac; Merck Life Science, Milan, Italy) 30 min before the addition of prostate CSCs. CFSE-labeled splenocytes from transgenic Rag-1^−/−^ OTI mice were co-cultured with irradiated CSCs in the presence of the synthetic peptide OVA_257−264_ (1 ng/ml) and 3.5 ng/ml IL-12 (R&D Systems) as previously described (38). After 4 or 3 days, respectively, cells were analyzed by FACS. Prostate-draining lymph nodes from TRAMP or WT mice were labeled with CFSE (30), cultured with or without 5 mM LacNAc, and analyzed after 3 days by FACS.

### Gal-3 Silencing

TPIN071122 cells were stably infected with Gal-3 shRNA Lentiviral Particles or with control shRNA Lentiviral Particles (Sigma) at 10 MOI, according to the manifacturer's protocol, to generate TPIN-SCshGal3#5 and TPIN-SCshScram, respectively. Briefly, 5 × 10^4^ cells/well were plated in a mixture of medium and Polybrene (Sigma). At day 2 lentiviral particles were added. At day 4 after infection, 2 μg/ml of puromycin dihydrochloride (Sigma) were added to select cells that had integrated the lentiviral particles.

### Tumor Challenge

2 × 10^6^ TPIN-SCshScram or TPIN-SCshGal3#5 were diluted 1:1 in Matrigel™ High Concentration (BD-Biosciences, Milan, Italy; 354248) and injected subcutaneously in male NSG recipients. 2 × 10^6^ TRAMP-C2 cells were injected subcutaneously in male C57BL/6N recipients. Mice were monitored twice weekly. Mice were sacrificed if the tumor became ulcerated. Tumor size was evaluated by measuring two perpendicular diameters and height by a caliper. Because tumors grew homogeneously as ellipsoid shaped masses, their dimension was calculated applying the ellipsoid volume formula: 4/3πabc, where a is height/2, b is width/2 and c is depth/2. To appreciate metastatic dissemination, the primary tumor was surgically resected when it achieved ≥ 80 mm^2^ (major diameter × minor diameter) (39). Mice were sacrificed when lymph node metastases were palpable, and ~1 month after surgery. Mice with no evidence of lymph node metastasis were killed 2 months after surgery.

### Flow Cytometry

Single cell suspensions were obtained from cell cultures, incubated 10 min with FcR blocker (BD-Biosciences), labeled for 15 min at 4°C with fluorochrome-conjugated monoclonal antibodies or isotype controls (all from BD-Biosciences or BioLegend), and acquired by BD FACSCanto™ as previously described (40). Dead cells were excluded by gating on 7AAD staining or based on physical parameters. For apoptosis test, samples were stained in Annexin V binding buffer (BD). Data were analyzed using FlowJo software.

### Western Blotting

Each cell pellet was homogenized in 10× volume of RIPA lysis buffer (10 mM Tris-Cl pH 7.2, 150 mM NaCl, 1 mM EDTA pH 8) with 1% Triton X-10/0.1% deoxycholate, 0.1% SDS, and protease and phosphatase inhibitor mixture (Roche). Samples were then diluted in Laemmli's SDS sample buffer. Proteins were separated by electrophoresis on 10% polyacrylamide gels according to the TGX Stain-Free FastCast Acrylamide kit protocol (Bio-Rad), and transferred onto Trans-Blot nitrocellulose membranes (Bio-Rad) according to the Trans-Blot Turbo Transfer System kit protocol (Bio-Rad). Ponceau staining (Sigma-Aldrich) was performed to confirm that the samples were loaded equally. The membranes were blocked in 5% non-fat dry milk in TBS-T (pH 7.4, with 0.1% Tween-20) for 1 h at room temperature. Primary antibodies were diluted in 3% BSA (Sigma-Aldrich) in TBS-T [mouse anti-calnexin 1:3,000 (Genetex) rat anti-mouse/human Gal-3 (E-Bioscience; 1:1,000)], and the membranes were incubated overnight at 4°C. The primary antibody was removed, and the blots were washed in TBS-T and then incubated for 45 min at room temperature in HRP-conjugated secondary antibodies [anti-mouse (Biorad) anti-rat (Amersham)]. Reactive proteins were visualized using a Clarity Western ECL substrate kit (Bio-Rad), and exposure was performed using UVItec (Cambridge MINI HD). Images were acquired by NineAlliance software.

### Gal-3 Knocking Out

Gal3- and Cd44-KO cell lines were generated using CRISPR/Cas9 technology. sgRNA targeting the coding sequence of lagls3 (CTCAAGGATATCCGGGTGCA) or Cd44 (GATGTAACCTGCCGCTACGC) were cloned into a modified version of the lentiCRISPR lentiviral vector plasmid (Zhang lab, Addgene #52961). This third-generation lentiviral vector backbone was generated by replacing both the existing promoter with a Spleen Focus Forming Virus (SFFV) promoter (41) and the puromycin cassette with an enhanced Green Fluorescent Protein (eGFP) selection transgene, and by inserting a loxP site in the 3′-self-inactivating Long Terminal Repeat. A scramble sequence against the murine genome (GATCGGCAAGGTGTGGGTCG) was used as negative control. Vesicular stomatitis virus glycoprotein G pseudotyped lentiviral vectors stocks were prepared as previously described (42). 10^5^ TPIN1323 were brought at single-cell suspension and then transduced at multiplicity of infection of 5. Transduction medium was changed after 24 h and cells were expanded. Fifteen days after LV transduction, eGFP positive cells were sorted at BD FACSAria III (BD Bioscience), obtaining up to 98% cell purityImaging flow cytometry. Knock-out of Gal3 and Cd44 were evaluated by Surveyor Assay (41) and flow cytometry, respectively.

### Imaging Flow Cytometry

The cells were resuspended at 40 × 10^6^ cells/ml incubated 10 min with FcR blocker (BD-Biosciences), labeled for 30 min at 4°C with fluorochrome-conjugated monoclonal mouse/human Gal-3 antibodies (1:25; E-Bioscience) just before acquisition. For surface staining, fresh cells were also stained with 7AAD to exclude dead cells just before acquisition. For intracellular staining, cells were fixed with 2% PFA and permeabilized with Triton-X (0.1% in PBS), before incubation with the desired antibodies. Gal-3 antibody (1:100). Fresh samples were imaged by ImageStream IS100 Imaging Flow Cytometer (Amnis, Merck) using a 40× objective. The excitation laser powers used were as follow: 488 (150 mW) and 658 (90 mW). Fixed cells were instead imaged by ImageStream X MarkII Imaging Flow Cytometer using a 40× objective. The excitation laser powers used were as follow: 405 (10 mW), 488 (200 mW), and 642 (20 mW). At least 10,000 events were collected in each sample, and single stained controls were acquired with identical laser settings to create compensation matrix. Data analysis was performed using the IDEAS software (Amnis). First of all, cells were gated for cells in focus using the gradient root mean square feature and then single cells were identified using area and aspect ratio features on the brightfield image. In fixed samples we evaluated the intracellular localization of Gal-3 protein. Single cells were gated for Dapi and Gal-3 double positivity, and nuclear localization of Gal-3 was assessed by Similarity feature in the nuclear region. Similarity feature is the log transformed Pearson's Correlation Coefficient and is a measure of the degree to which two images are linearly correlated within a masked region (IDEAS software). Thus, when the intensity of Gal-3 in the nuclear region is high and Dapi staining is high, there is a linear correlation between the two images and the similarity feature has a high positive value.

### Real-Time PCR

Total RNA from CSCs was extracted using the RNeasy Plus Mini kit (Qiagen, Chatsworth, CA, USA). cDNA was obtained from 1,000 ng of RNA using the M-MLV-Reverse Transcriptase kit (Promega, Madison, WI, USA). Real-Time PCR was performed in a total volume of 20 μL using the Taqman® Universal PCR Master Mix (Applied Biosystems, Monza, Italy), 2 μL of cDNA (prediluted 1:1) and specific probes for Gal-3 or L-19 (Applied Biosystems, Italy). Values were normalized to internal control (L-19) using the ΔCT method.

### Immunohistochemistry and Immunofluorescence of Human and Mouse Samples

After institutional review board approval, a cohort of nine patients with pelvic node-positive prostate cancer treated with radical prostatectomy and extended pelvic lymph node dissection were randomly selected from our prospectively collected data-base. Human and TRAMP prostate or lymph node specimens were embedded in paraffin. Five micrometer sections were stained with Mayer- Hematoxylin and Eosin (BioOptica, Milan, Italy) and evaluated by an expert pathologist (33). Alternatively, after re-hydratation, antigen retrieval in 10 mM citric acid and blocking with 5% NGS, slides were incubated with the anti mouse/human Gal-3 (1:200; E-Bioscience) overnight at 4°C. A biotinylated secondary antibody was used 1:250 for 1 h at room temperature. Colorimetric revelation was made with Novared chromogen (Vector Labs, Burlingame, CA, USA). Slides were finally contrasted with Mayer-Hematoxylin (BioOptica), mounted with cover glass and examined under microscope (Carl Zeiss, Axioscope 40FL, Varese, Italy). Prostate CSCs or prostate cancer cells were plated on a matrigel-coated glass slide overnight, fixed with 4% PFA and permeabilized with PBS containing 0.1% Triton-X. After blocking with Triton X-100 0.1 and 5% NGS for 1 h at room temperature, cells were incubated with rat anti-mouse/human Gal-3 (E-Bioscience; 1:200) for 2 h at room temperature, and then with anti-rat Alexa 546 secondary antibody (E-bioscience; 1:200). Nuclei were stained with 0.1 μg/mL DAPI and slides were examined under TCS SP2 confocal microscope (Leica, Milan, Italy). For Oct-4 staining prostate CSCs were plated on a matrigel-coated glass slide overnight, fixed with 4% PFA and permeabilized with PBS containing 0.1% Triton-X and 2% BSA. After blocking with Triton X-100 0.1 and 5% NGS for 1 h at room temperature, cells were incubated with rat anti-mouse/human Oct-3/4 (Santa Cruz Biotechnology; 1:20) and rabbit anti-GFP (Invitrogen; 1:250) for 2 h at room temperature, and then with PE anti-mouse secondary antibody (Santa Cruz Biotechnology; 1:200) and anti-rabbit Alexa 488 secondary antibody (Life Technologies; 1:500), respectively. Nuclei were stained with 0.1 μg/mL DAPI and slides were examined under TCS SP2 confocal microscope (Leica, Milan, Italy).

### IncuCyte

Cell and spheroid proliferations were assessed using Incucyte (Sartorius Essen Biosciences, AnnArbor, MI) over 88 h in culture, with image capture every 4 h. Cell and spheroid proliferations were measured and reported as mean area confluence percentage, IncuCyte Image Analysis Software. Spheroids identification was allowed by setting a minimum dimension in order to distinguish them from single cells.

### Microarray-Based Gene Expression Profiling

Total RNA extracted using the RNeasy Micro and Mini kit (Qiagen, Chatsworth, CA, USA) was analyzed with Affimetrix Mouse Gene 1.0 ST Array as previously described (29).

### Statistical Analyses

Statistical analyses were performed using the Student's *T*, One-Way Anova followed by Tukey's tests, or Fisher exact test. Values were considered statically significant for ^*^p < 0.05, ^**^p < 0.01; ^***^p < 0.001, ^****^p < 0.0001.

## Results

### Gal-3 Favors Proliferation, Spheroid Formation, Tumorigenicity, and Metastatic Potential of TPIN-SCs

**Figure 1:**
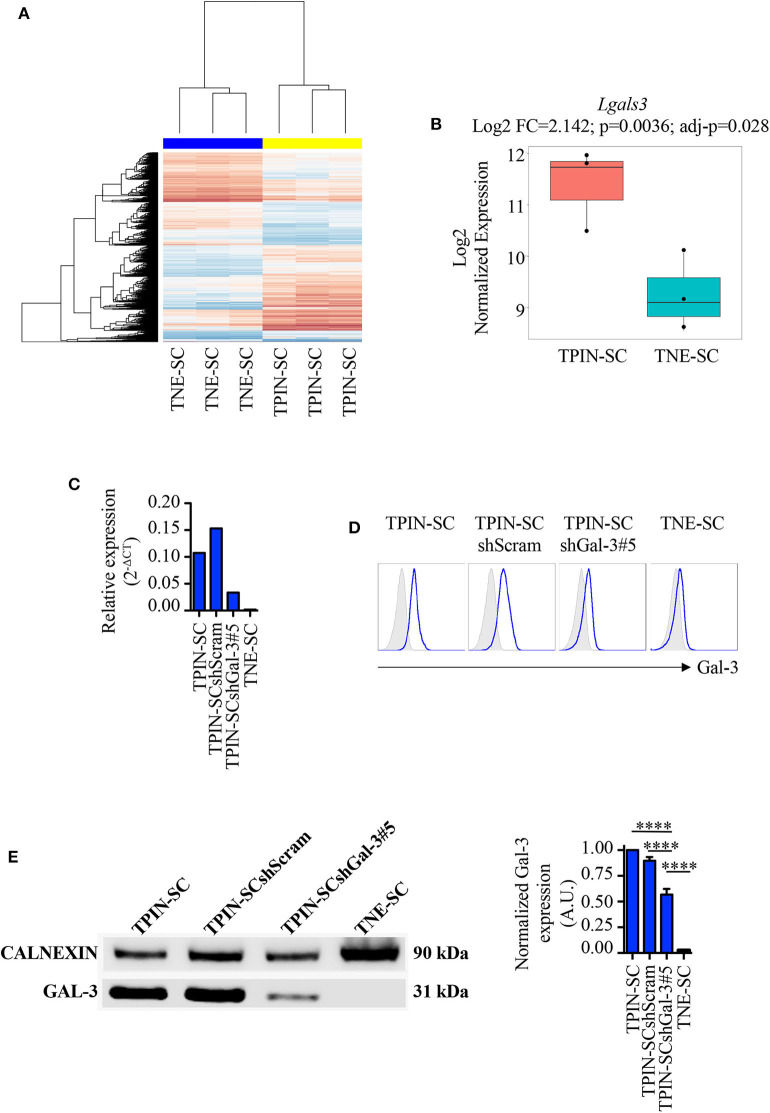
Gal-3 is overexpressed in TPIN-SCs, and can be silenced by shRNA technology. Gene expression analysis of CSCs with Affimetrix Mouse Gene 1.0 ST Array. **(A)** Heatmap reports the global gene expression data in TPIN-SCs vs. TNE-SCs. Differentially expressed genes with a *p* < 0.05 are red (upregulated) or blue-colored (downregulated). **(B)** Boxplot reports Lgals3 expression in TPIN-SCs vs. TNE-SCs (Log2 FC = 2.142, *p* = 0.0036, *adj-p* = 0.028). Gal-3 silencing in TPIN-SCs was attempted with five different shRNA sequences. We obtained substantial inhibition of Gal-3 expression in all TPIN-SCs infected with viruses encoding Gal-3 specific shRNA (not shown). We selected TPIN-SCshGal-3#5 for our experiments. Expression of Gal-3 in the indicated cells was assessed by real-time PCR **(C)**, flow cytometry **(D)** and Western blot **(E)** analysis. **(C)** Relative expression of Gal-3 in the indicated cells was assessed by real-time PCR. **(D)** Fresh samples for cell surface detection of Gal-3 were stained with anti-Gal-3 antibody and 7AAD. The plots report representative histograms of Gal-3 staining (blue lines), gray histograms: isotype control. TNE-SCs were used as negative control for Gal-3 expression. The panel is representative of at least three independent experiments. **(E)** Western blotting analysis of total Gal-3 expression in the indicated cell lines and relative quantification. The Western blot is representative of two independent experiments, performed each time on biological duplicates. The graph is a pool of four independent blots. Statistical analysis was performed using the Anova Test. ****p < 0.0001.

We have previously reported that TPIN-SCs, despite their highly metastatic behavior (43), can be targeted both by innate and adaptive immune cells (44), but are also endowed with immunosuppressive activities (45). In particular, we have found that TPIN-SCs use the extracellular matrix protein Tenascin-C to dampen T cell activation (30, 46), a mechanism that might favor their metastatic propensity. However, Tenascin-C silencing in TPIN-SC did not completely abolish their immunosuppressive activity, thus suggesting that additional molecules were involved in CSCs-mediated immunosuppression (30). In search for additional mechanisms favoring CSC aggressiveness, we mined data from transcriptomic analyses of TPIN-SCs and CSCs derived from neuroendocrine tumors (TNE-SCs; Figure 1A) (29, 30), the latter being devoid of immunosuppressive activity (30). We found that lgals3, the gene coding for Gal-3, was over-expressed in TPIN-SCs (Figure 1B). Gal-3 expression in TPIN-SCs was confirmed by real time PCR (Figure 1C), flow cytometry (Figure 1D), and western blot (Figure 1E).

**Figure 2:**
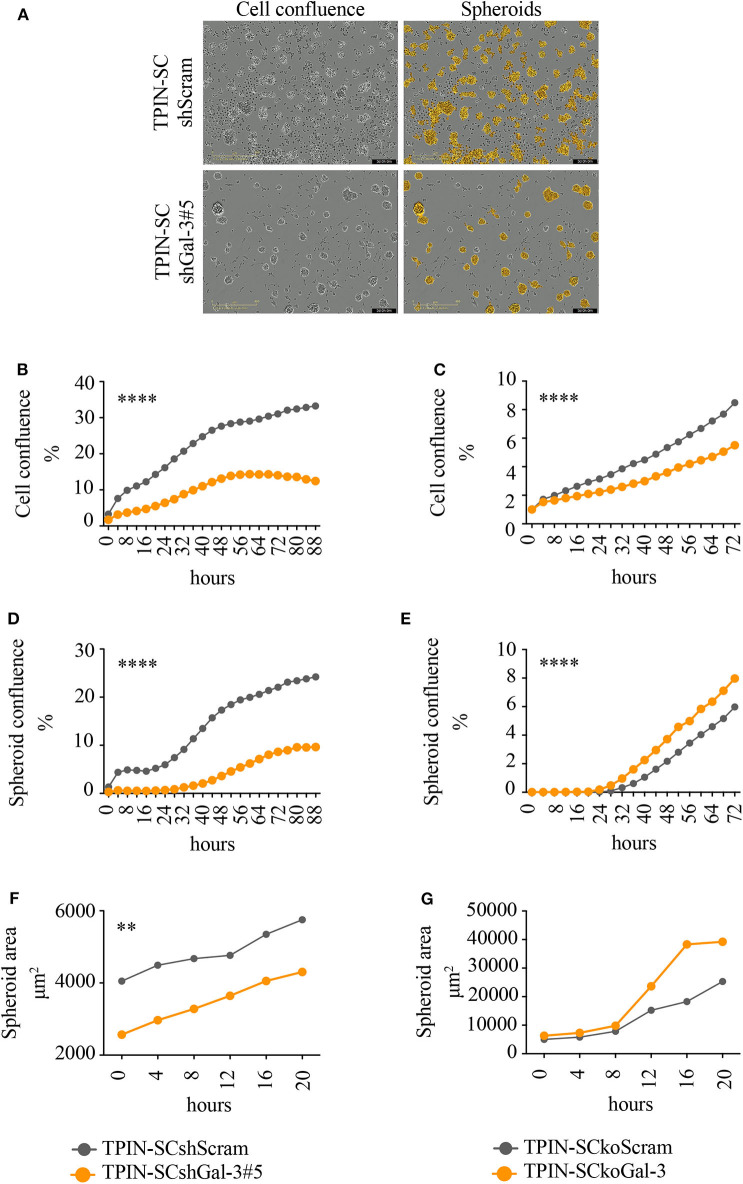
Gal-3 impacts TPIN-SC proliferation, and sphere formation, tumorigenicity, and metastatic potential. TPIN-SCshScram or TPIN-SCshGal-3#5 were analyzed by IncuCyte for 88 h consecutively **(A,B,D,F)**, and TPIN-SCkoGal-3 and TPIN-SCkoScram were analyzed 72 h consecutively **(C,E,G)**, with image captured every 4 h. **(A)** Representative images of cell confluence and spheroids at day 3. Orange: mask to identify spheroids; objective: 10×; scale: 400 μm. **(B,C)** Cell proliferation was measured and reported as mean cell confluence percentage. **(D,E)** Spheroid proliferation was measured and reported as mean spheroid confluence percentage. **(F,G)** Spheroids dimension at day 3 was measured and reported as mean spheroid area (μm^2^). Statistical analyses were performed using the Student *T*-test. **p < 0.01; ****p < 0.0001.

To assess the role of Gal-3 in CSC biology, we infected TPIN-SCs with lentiviral vectors encoding either a Gal-3-specific or a scrambled short hairpin RNA (shGal-3#5 and shScram, respectively). qPCR, flow cytometry and western blot analyses (Figures 1C–E) confirmed Gal-3 silencing in prostate CSCs. Strikingly, Gal-3 silencing in TPIN-SCs reduced cell confluence (Figures 2A,B) and spheroid confluence and area (Figures 2A,D,F). These results, although consistent with the hypothesis that Gal-3 regulates proliferation and sphere formation in CSCs, were not conclusive because Gal-3 expression in TPIN-SCshGal-3#5 cells was not totally abolished (Figure 1). Thus, we generated TPIN-SCkoGal-3 cells by knocking out lgals3 in TPIN-SCs (Supplementary Figure 1), and we compared their proliferation and sphere formation capacity with TPIN-SCkoScram (Supplementary Figure 1). As shown in Figure 2C, TPIN-SCkoGal-3 proliferated less than TPIN-SCkoScram, thus confirming that Gal-3 is important for TPIN-SC proliferation. At difference with TPIN-SCshGal-3#5 cells, TPIN-SCkoGal-3 did not show a reduced spheroid confluence and area (Figures 2E,G). Indeed, spheroid confluence was higher in TPIN-SCkoGal-3 than in TPIN-SCkoScram. A direct comparison between TPIN-SCshGal-3#5 and TPIN-SCkoGal-3 cannot be done because they have been derived from two different CSCs lines. Nevertheless, data reported in Figure 2 suggest that Gal-3 dynamically regulate both cell proliferation and cell adhesion. Indeed Gal-3 contributes to cell-cell and cell-matrix interactions (8). Thus, it can be hypothesized that in the absence of endogenous Gal-3, less proliferating CSCs remain in clusters. CSCs might also compensate lack of Gal-3 by upregulating expression of other molecules involved in spheroid formation.

**Figure 3:**
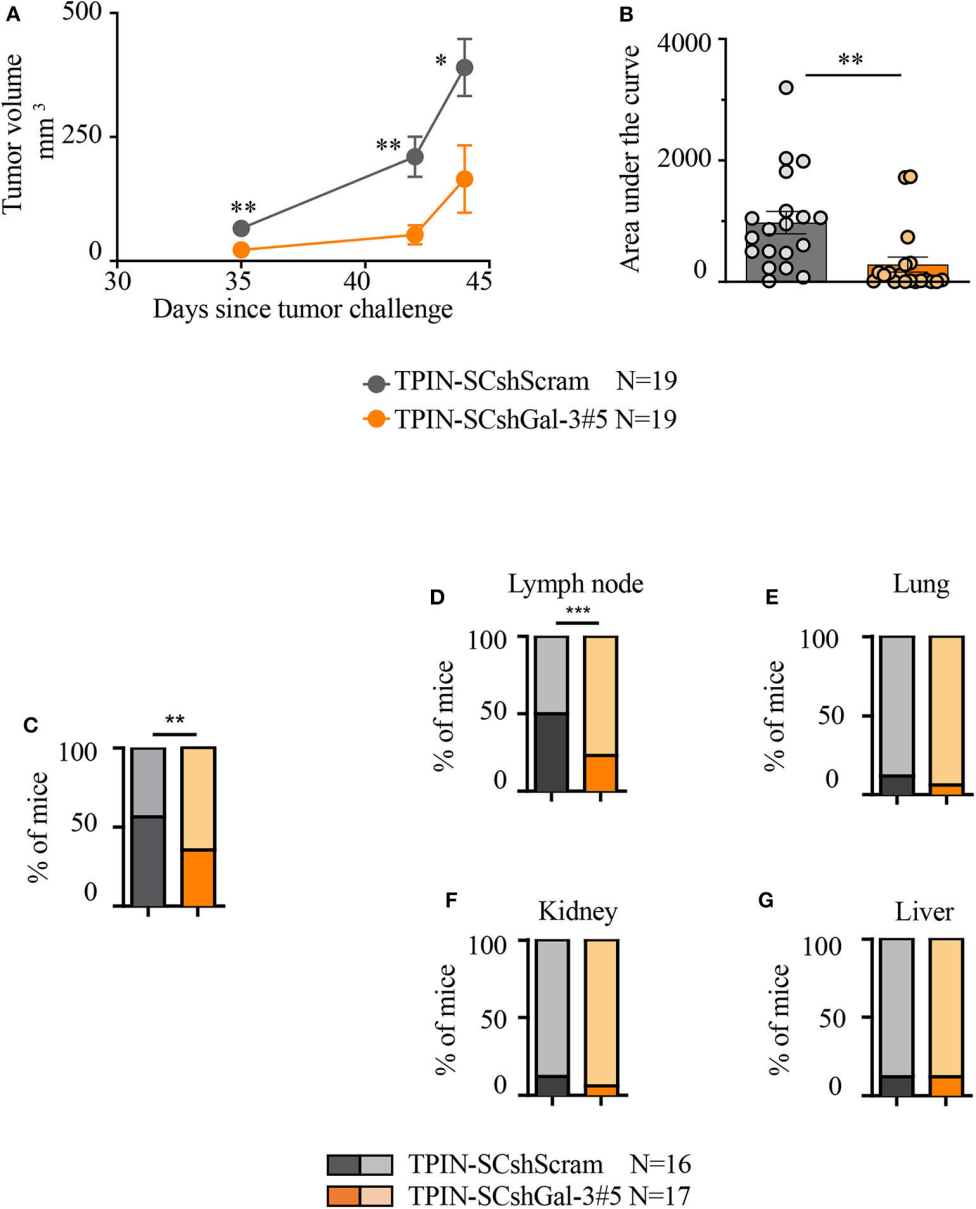
Gal-3 impacts TPIN-SC tumorigenicity and metastatic potential. **(A)** Immunodeficient NSG mice received 2 × 10^6^ TPIN-SCshScram or TPIN-SCshGal-3#5 (19 mice/group). The graph reports tumor growth (mm^3^) progression volume. Average ± SEM of tumor volume. **(B)** The graph reports tumor progression expressed as area under the curve at day 44. Data are reported as a percentage ± SEM. Statistical analysis was performed using the Student *T*-test. Data represent a pool of four independent experiments. **(C)** When tumor area achieved ≥80 mm^2^, the primary tumors were surgically resected to monitor metastatic spreading. The graph reports TPIN-SCshScram or TPIN-SCshGal-3#5 metastatic ability. **(D–G)** The graphs report the percentage of lymph node **(D)**, lung **(E)**, kidney **(F)**, liver **(G)**, metastatic spreading. Dark colors indicate metastasis-bearing mice, whereas light colors indicate metastasis-free mice. TPIN-SCshScram: *n* = 16 mice; TPIN-SCshGal-3#5: *n* = 17 mice. Statistical analysis was performed using the Fisher Exact Test. *p < 0.05; **p < 0.01; ***p < 0.001.

To investigate the tumorigenic and metastatic potential of prostate CSCs in a context devoid of the potentially confounding effects of the immune system, immunodeficient NSG mice were challenged with TPIN-SCs either silenced or not for Gal-3. TPIN-SCshScram and TPIN-SCshGal-3#5 generated tumors in 100% of NSG mice, but tumor growth was delayed in mice challenged with TPIN-SCshGal-3#5 (Figure 3A), and at day 44, the tumor dimension was reduced in mice challenged with TPIN-SCshGal-3#5 when compared to mice challenged with TPIN-SCshScram (Figure 3B). These data demonstrate that Gal-3 has a relevant tumor-cell intrinsic effect on prostate cancer progression.

To investigate the metastatic potential of TPIN-SCs, the primary subcutaneous lesion was surgically resected when the tumor mass reached an area of ≥80 mm^2^, and mice were monitored thereafter for metastasis occurrence. The primary lesion was excised to allow metastases to show up before mice had to be culled due to primary lesion overgrowth. Gal-3 silencing in TPIN-SCs reduced their metastatic potential. Indeed, metastases were found in 56% of the mice injected with TPIN-SCshScram, and only in 36% of TPIN-SCshGal-3#5-challenged mice (Figure 3C). Tumor-draining lymph nodes were the preferred site of invasion upon subcutaneous injection. Interestingly, Gal-3 silencing in TPIN-SCs significantly reduced their tropism for lymph node invasion, and metastatic lymph nodes were found in 24% of the mice injected with TPIN-SCshGal-3#5, and 50% of mice challenged with TPIN-SCshScram (Figure 3D). Lack of Gal-3 did not significantly impact metastatic appearance in the lungs (Figure 3E), kidneys (Figure 3F), and liver (Figure 3G). Altogether, these findings suggest that Gal-3 has a relevant tumorigenic and metastatic role in prostate CSCs.

### Localization of Gal-3 in Prostate CSCs and in TRAMP-C2 Prostate Cancer Cells

**Figure 4:**
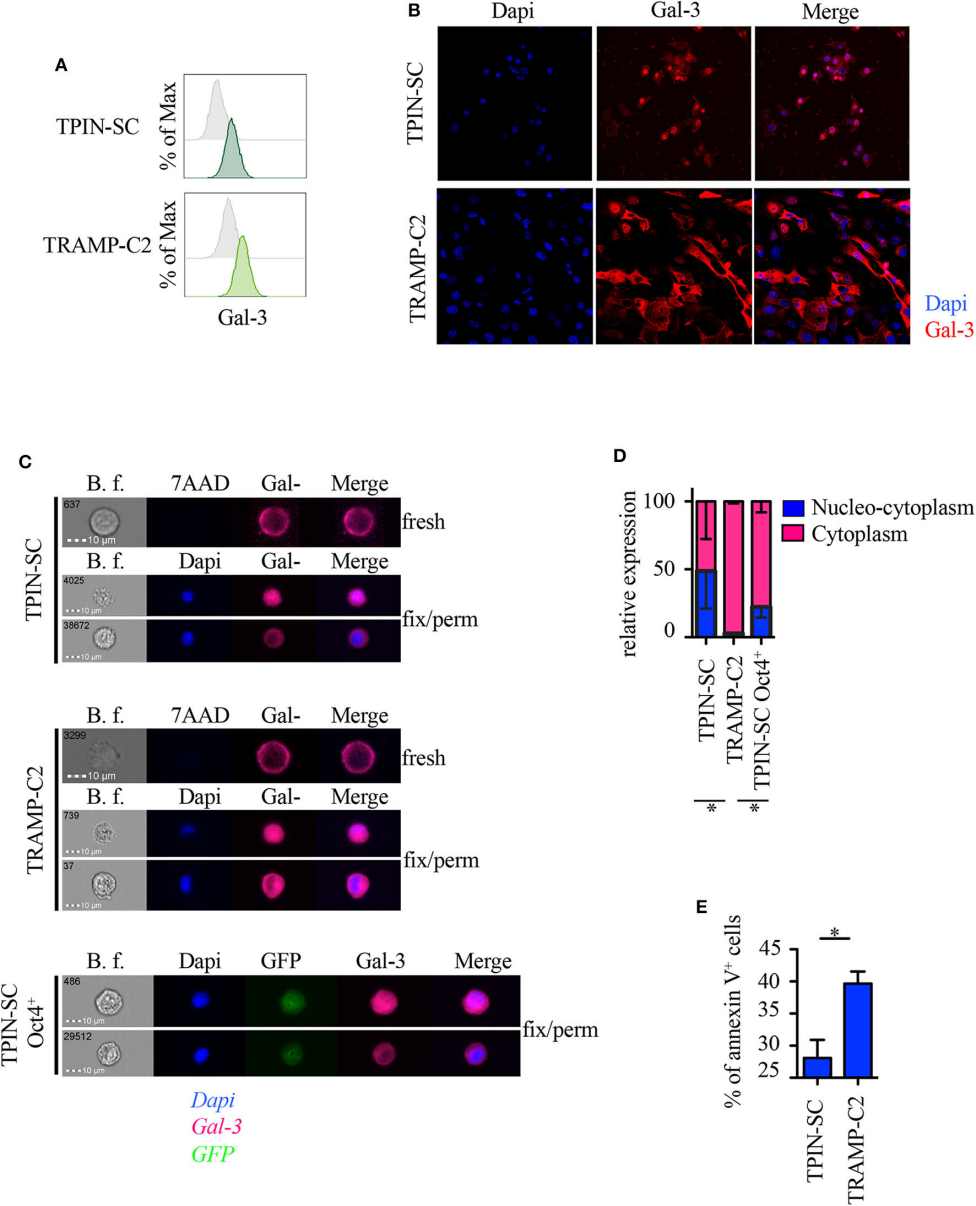
Gal-3 is differently distributed in prostate CSCs and in more differentiated cancer cells. Expression of Gal-3 in the indicated mouse cells was assessed by flow cytometry analysis **(A)**, immunofluorescence **(B)** and ImageStream technology **(C)**. Fresh samples for cell surface detection of Gal-3 **(A,C)** were stained with 7AAD, while fixed and permeabilized samples for intracellular detection of Gal-3 were stained with Dapi **(B,C)**. In fresh samples for cell surface detection of Gal-3, dead cells were excluded by 7AAD positivity. Since we analyzed live cells only (thus, 7AAD negative), **(C)** does not show any 7AAD positivity. Cells were also stained with anti-Gal-3 antibodies. **(A)** Representative histograms of Gal-3 staining (green lines). Gray histograms: unstained. **(B)** Representative confocal images of Gal-3 staining. Red: Gal-3; Blue: Dapi. Magnification 63×. Images were optimized for brightness/contrast using imageJ. **(C)** Representative ImageStream images of Gal-3 staining. Magenta, Gal-3; Blue, Dapi; Green, GFP. **(D)** Quantification of Gal-3 intracellular distribution by ImageStream technology. Data are reported as relative expression of Gal-3 among the indicated cell lines; the blue bar reports the percentage of nucleo-cytoplasmic distribution of Gal-3, the magenta bar reports the percentage of mainly cytoplasmic distribution of Gal-3. Statistical analysis was performed using Student *T*-test. The panel is a pool of three independent experiments. **(E)** Quantification of Annexin V^+^ cells analyzed by flow cytometry in the reported cell lines. Statistical analysis was performed using Student *T*-test. The panel reports one experiment representative of three independent experiments. *p < 0.05.

Because subcellular Gal-3 localization correlates with its function in tumor cells (10), we analyzed prostate CSCs by flow cytometry, immunofluorescence, and imageStream technology, which combines flow cytometry with the detail imagery of microscopy. TPIN-SCs expressed Gal-3 at the cell surface (Figure 4A) and in the intracellular space (Figures 4B,C). Approximately 50% of the TPIN-SCs were Gal-3 positive both in the cytoplasm and in the nucleus, whereas the remaining 50% showed a preferential cytoplasmic localization (Figures 4C,D). Also TRAMP-C2 cells, a line of more differentiated prostate cancer cells obtained from a TRAMP adenocarcinoma (34), expressed Gal-3 (Figures 4A–C). However, the majority of TRAMP-C2 cells showed a preferential cytoplasmic localization of Gal-3 (Figures 4B–D). Thus, Gal-3 is differently distributed in TPIN-SCs and more differentiated prostate cancer cells.

It has been recently reported that Gal-3 promotes lung cancer stemness via the EGFR/c-Myc/Sox-2 pathway (24), and Oct4, a stemness-related transcription factor (47), favors Gal-3 expression, thus establishing a positive regulatory loop in lung CSCs (24). To investigate whether a different intracellular expression of Gal-3 associates with different functional states (i.e., stem cells and their differentiated progeny), we transduced prostaspheres generated from TPIN-SCs, which contained both CSCs and committed more differentiated precursors, with a lentiviral vector carrying the GFP sequence under the control of the Oct4 promoter (48). Thus, only Oct4^+^ cells expressed GFP. GFP^+^ cells were sorted by flow cytometry to obtain a pure population of GFP^+^ TPIN-SCs. Upon *in vitro* culture, GFP^+^ TPIN-SCs progressively gave rise to ~20% GFP^−^ TPIN-SCs, thus suggesting that the CSC core of GFP^+^ TPIN-SCs autonomously and progressively re-establishes the heterogeneity found in prostaspheres, allowing some CSCs to differentiate by decreasing Oct4 expression (Supplementary Figures 2A,B). We focused on Oct4^+^ TPIN-SCs, as they likely constitute highly undifferentiated CSCs. By ImageStream technology, we found that Oct4^+^ TPIN-SCs had a pattern of intracellular Gal-3 distribution comparable to TPIN-SCs (Figures 4C,D). We conclude that TPIN-SCs are composed of CSCs that *in vitro* spontaneously generate a population of committed more differentiated precursors. Gal-3 has a preferential cytoplasmic and nuclear localization in TPIN-SCs, irrespective of their differentiation stage.

Because Gal-3 may favor or protect from apoptosis depending on its intracellular localization (10), we investigated apoptosis in TPIN-SCs and the more differentiated TRAMP-C2 cells. TPIN-SCs were less prone to undergo apoptosis than TRAMP-C2 cells (Figure 4E, Supplementary Figure 3), thus suggesting that nucleus-cytoplasmic distribution of Gal-3 in CSCs protect them from apoptosis.

### TPIN-SCs Use Gal-3 to Dampen T Cell Proliferation

**Figure 5:**
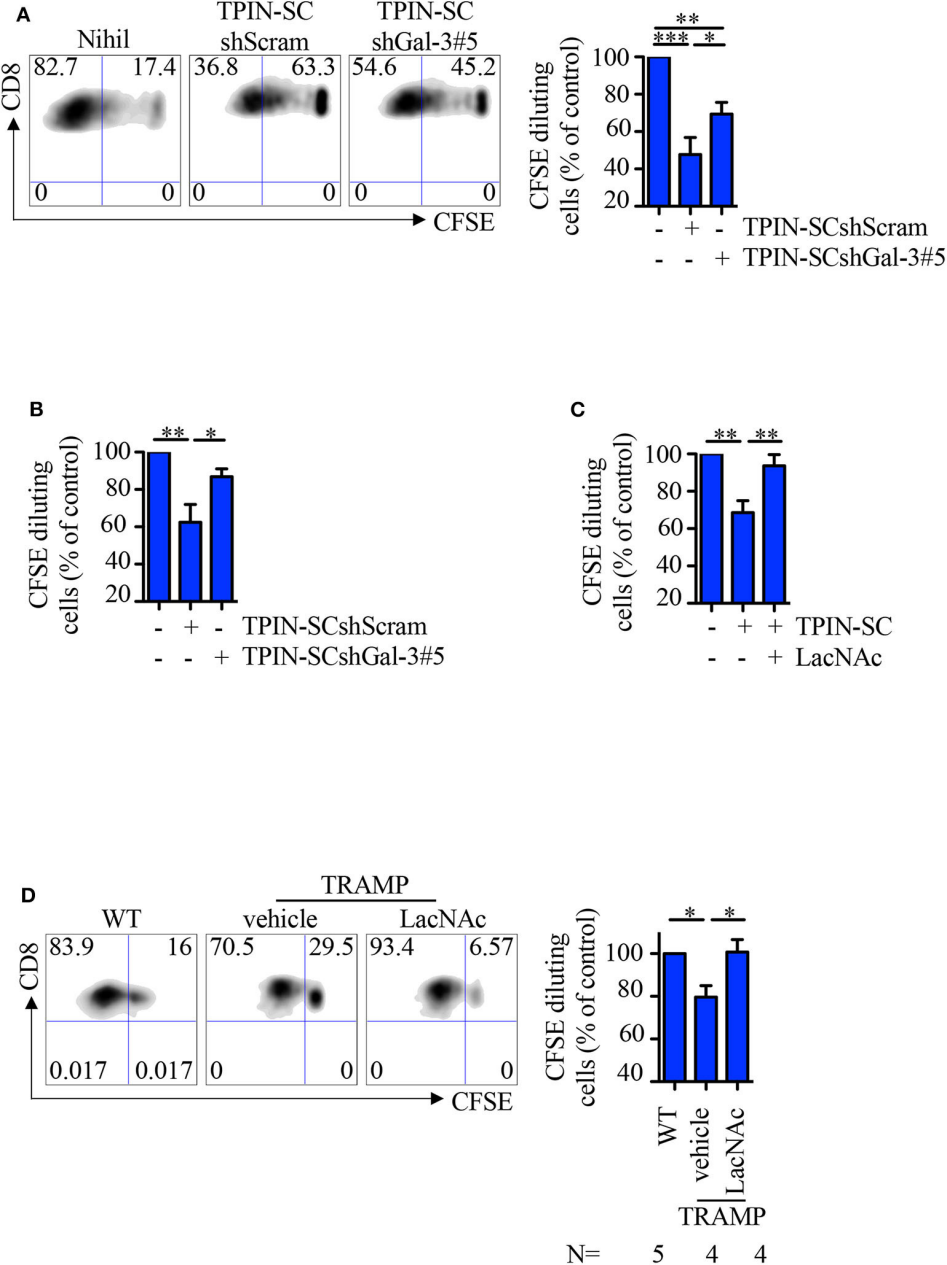
TPIN-SC use Gal-3 to dampen T cell proliferation. Naïve **(A,C)** or RAG-OT1 **(B)** splenocytes were labeled with CFSE, and activated with anti-CD3 and anti-CD28 beads **(A,C)** or OVA **(B)**, respectively, in the presence or absence of irradiated TPIN-SC cells **(C)**, or TPIN-SC infected with lentiviral vectors encoding Gal-3-specific shRNA [TPIN-SCshGal-3#5 **(A,B)**] or unspecific [TPIN-SCshScram **(A,B)**]. Where indicated, 5 mM LacNAc was added to the culture **(C)**. Representative dot plots of CFSE dilution for each experimental condition **(A)** and quantification of CFSE dilution reported as percentage of CD8 proliferating T cells at day 4 **(A,C)** or day 3 **(B)**. Values were normalized to the positive control (splenocytes activated with anti-CD3 and anti-CD28 beads or OVA). Statistical analysis was performed using Anova followed by Tukey's test or Student *T*-test. Graph **(A)** is a pool of nine independent experiments; graph **(B)** is a pool of four independent experiments; graph **(C)** is a pool of six independent experiments. **(D)** CFSE dilution of CD8 T cells from prostate-draining lymph nodes of 12/13-week-old TRAMP and wild type (WT) mice at day 3 of stimulation with anti-CD3/CD28 beads. Cells from TRAMP-derived prostate-draining lymph nodes were subdivided in two parts, one of which was also incubated with 5 mM LacNAc. Representative dot plots of CFSE dilution for each experimental condition, and quantification of the percentage of CD8 proliferating T cells at day 3. Each panel is representative of at least two independent experiments. Values were normalized to the positive control (WT). Statistical analysis was performed using Anova followed by Tukey's test. The graph is a pool of four independent experiments. *p < 0.05; **p < 0.01; *** *p*<0.001.

Because Gal-3 is immunosuppressive (11), we asked if TPIN-SCs utilize Gal-3, together with Tenascin-C (30), to suppress T cell-mediated immune responses. To this aim, CD8 T cells from the spleen of naive or TCR transgenic RAG-OT1 mice were labeled with CFSE, and stimulated with anti-CD3/CD28 beads or the OVA_257−264_ peptide, respectively, in the presence of prostate CSCs. Whereas, as expected (30), the addition of TPIN-SCshScram to the culture blocked T cell proliferation, Gal-3 silencing in TPIN-SCs substantially dampened their immunomodulatory effects (Figures 5A,B). The direct immunomodulatory role of Gal-3 was confirmed by adding the Gal-3 synthetic inhibitor LacNAc (49) to the co-culture with TPIN-SCs, and showing that CD8 T cell proliferation was rescued (Figure 5C).

We previously showed that CSCs precociously migrate to prostate-draining lymph nodes of TRAMP mice affected by PIN through the CXCR4/CXCL12 axis, and participate in generating a local immunosuppressive microenvironment (30, 46). Indeed, CD8 T cells obtained from prostate-draining lymph nodes of TRAMP mice proliferated less than T cells from prostate-draining lymph nodes of age-matched wild type littermates (Figure 5D). To investigate if Gal-3 was responsible for this phenomenon, CFSE-labeled naïve cells from prostate-draining lymph nodes of TRAMP mice were cultured with anti-CD3/CD28 beads and LacNAc (Figure 5D). Flow cytometry analysis of CD8 T cells showed that in the presence of LacNAc, T cell proliferation was restored to the levels of T cell proliferation in age-matched wild type mice, thus confirming that Gal-3 contributes to the immunosuppressive milieu in TRAMP lymph nodes. Altogether, these findings suggest that Gal-3 participates to the immunosuppressive activity of TPIN-SCs both in the primary tumor lesion and in precociously invaded lymph nodes.

### Gal-3 Is Expressed in Human and Mouse PIN Lesions and Metastatic Lymph Nodes

**Figure 6:**
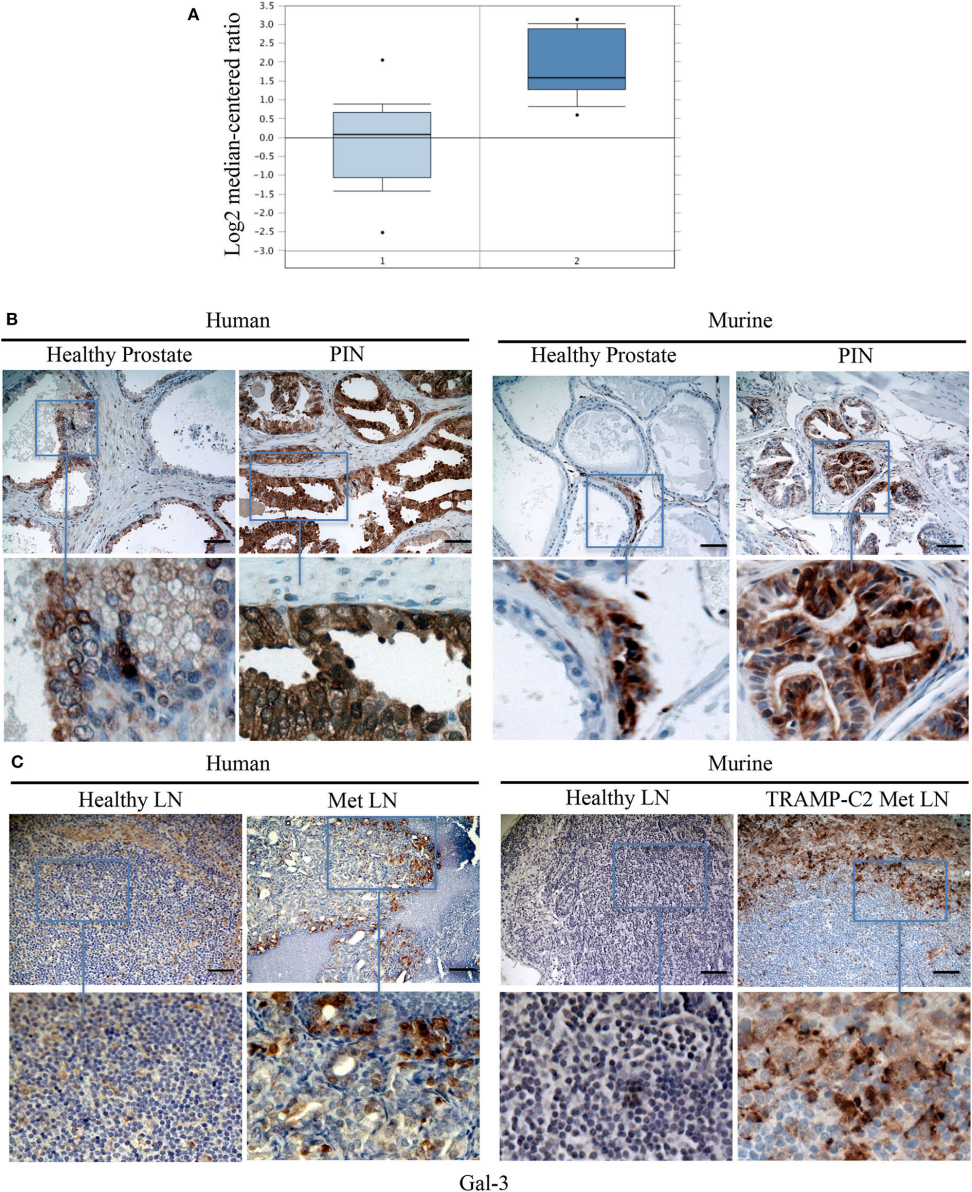
Gal-3 is expressed in human and mouse PIN lesions and metastatic lymph nodes. **(A)** The levels of Gal-3 transcripts in normal and cancerous human prostate carcinoma tissues. Raw data were retrieved from the Oncomine (www.oncomine.org), Tomlins Prostate Dataset (50). Transcript level of Gal-3 in PIN samples (2), *n* = 13, was compared with samples prostate gland (1), *n* = 23. Box plot represents the median with 90th and 10th percentiles, and statistical significance was analyzed by Student *T*-test, *p* = 0.00307. Formalin fixed paraffin embedded sections from human and mouse: healthy prostate and PIN lesions **(B)** or healthy and metastatic (Met) lymph nodes (LN) **(C)** were stained by immunohistochemistry in order to evaluate Gal 3 expression. Scale Bar = 20 mm. In blue boxes zoom details. Slides are representative of at least three different cases. Images were optimized for brightness.

Because TPIN-SCs originate from PIN lesions, we searched for a published gene signature of human normal prostate and PIN (50). As reported in Figure 6A, the Gal-3 transcript was found overexpressed in human PIN when compared to healthy prostate. We next validated Gal-3 expression at the protein level by immunohistochemistry. While in the healthy human and mouse prostate, Gal-3 showed a weak immunostaining primarily localized in the cytoplasm (Figure 6B), in human PIN lesions Gal-3 staining was rather heterogeneous and intense, and mainly cytoplasmic (Figure 6B), thus confirming previous findings (14). Interestingly, Gal-3 was also found in mouse PIN lesions, where it showed a patchy distribution (Figure 6B). Thus, both human and murine PIN lesions express Gal-3 preferentially in the cytoplasm of transformed cells, as we found in differentiated TRAMP-C2 cells (Figure 4).

Based on our findings in the TRAMP model (Figure 3), and the known role of Gal-3 in the metastatic process (51), we were interested in investigating the expression of Gal-3 in metastatic prostate cancer. To this aim, we stained with anti-Gal-3 antibodies human Du145 cells, which were derived from a central nervous system metastasis (35), and PC3 cells obtained from a metastatic lymph node (36). At flow cytometry, both cell populations clearly expressed Gal-3 (Supplementary Figure 4). By immunohistochemistry we found that Gal-3 was strongly expressed in metastatic bone from prostate cancer patients (Figure 6C and Table 1), thus confirming previous findings (52). We also originally observed that in some samples Gal-3 staining was stronger at the invading edge of lymph node metastasis (Figure 6C and Table 1).

**Table 1:** Gal-3 expression in tumor cells invading metastatic lymph nodes.

	**Gal-3^**+**^samples (%)**	**Gal-3 at leading edge (%)**
Human	9/9 (100)	2/9 (22)
Mouse	8/13 (62)	2/8 (25)

*Number of human and mouse metastatic lymph nodes stained for Gal-3 by immunohistochemistry. Human samples: pelvic lymph nodes from 9 patients. Murine samples: axillary/inguinal lymph nodes from 7 mice challenged with TRAMP-C2 cells. Leading edge refers to intense Gal-3 staining in cancer cells at the interface with lymphocytes in lymph nodes*.

We also investigated Gal-3 expression in mouse lymph nodes affected by measurable metastasis. The incidence of measurable lymph node metastasis in TRAMP mice has been reported to be very low (30, 53). A recent survey in our colony of 88 TRAMP mice found lymph node metastases by adenocarcinoma, which were confirmed by the pathologist, in two mice, accounting for ~3% of the screened animals. To overcome the limitation of the autochthonous TRAMP model, we took advantage of the well-established model of lymph node metastasis upon subcutaneous challenge with TRAMP-C2 cells (39). Thus, C57BL/6 mice were challenged with TRAMP-C2 cells (Supplementary Figure 5). When the tumor area reached ≥80 mm^2^, we surgically resected the primary tumor, and monitored mice for lymph node metastasis occurrence. Approximately 1 month after surgery, 86% of the mice (i.e., six out of seven mice) developed axillary and inguinal lymph node metastases. In 62% of the metastatic lymph nodes from TRAMP-C2-challenged mice we found Gal-3 expression in neoplastic cells invading the lymph node (Table 1). Similarly to the human counterpart, 25% of metastatic lymph nodes from TRAMP-C2-challenged mice had a more intense Gal-3 staining at the invading edge of the metastasis (Figure 6C and Table 1). Altogether, these findings demonstrate that Gal-3 is expressed in human and murine PIN lesions as well as in metastatic lymph nodes.

## Discussion

Prostate cancer is one of the most frequently diagnosed cancers, and it accounts for 19% of all estimated new cancer cases in men (54). Metastatic dissemination is a severe complication of prostate cancer, and the main cause of cancer mortality. The majority of prostate cancer patients harbor bone with lymph-node metastases, 6% develop exclusive lymph node disease recurrence and 20% have visceral metastases (55). When prostate cancer becomes castration-resistant it is essentially incurable. Indeed, prostate cancer is one of the major causes of death by cancer and accounts for 9% of estimated cancer deaths in men (54). Thus, a better understanding of the metastatic process in prostate cancer is essential to direct current and future therapeutic strategies.

**Figure 7:**
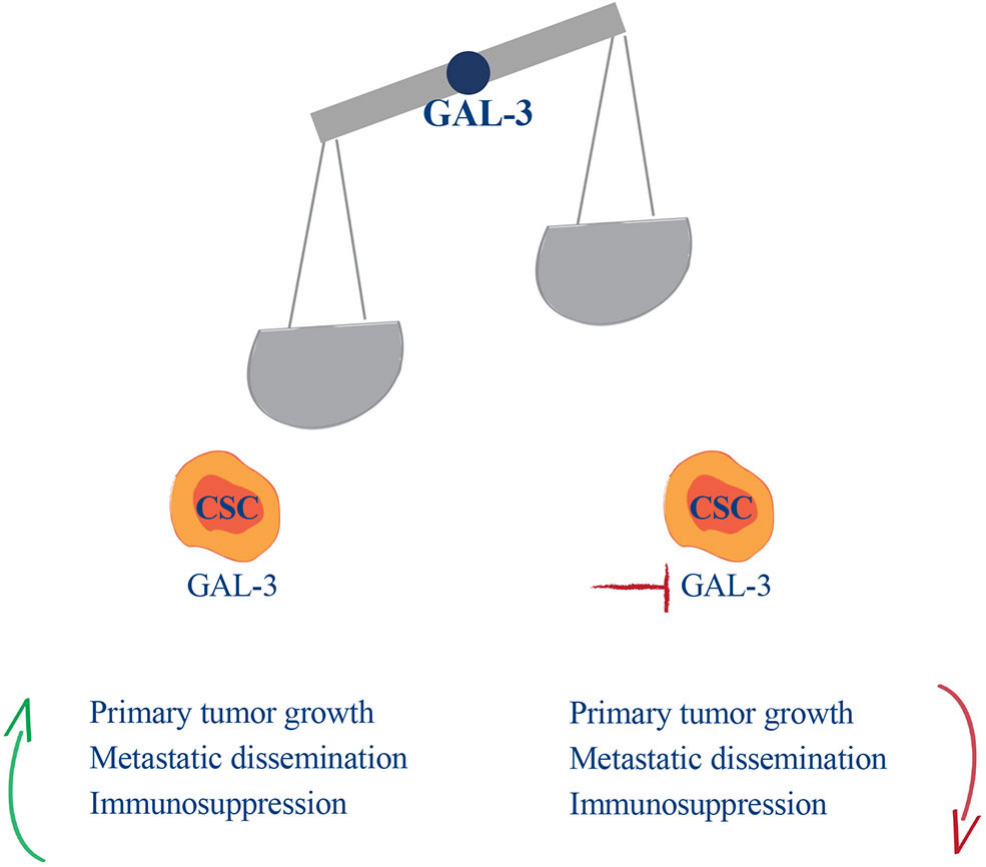
Gal-3 balances cancer malignancy in the early phases of prostate cancer development. In the early phases of disease development and metastasis CSCs, through Gal-3, drive prostate cancer malignancy, and immunosuppression.

The prostate cancer microenvironment is immunosuppressive (56, 57). We and others have previously reported that in TRAMP mice, especially in the early phases of cancer development and progression (33, 58), the tumor microenvironment is endowed with redundant immunosuppressive mechanisms. These are operated by several cell populations, including regulatory T cells (59, 60), myeloid derived suppressor cells (61, 62), and prostate CSCs (30). Our new findings suggest that Gal-3 is an additional mechanism of immune suppression that acts both in primary prostate lesions and in lymph nodes. Gal-3 also exerts pro-metastatic functions in CSCs. Several experimental evidences support our conclusions. Firstly, Gal-3 expressed in TPIN-SCs dampened T cell proliferation, and Gal-3 silencing in TPIN-SCs or the addition of LacNac to the co-culture recued T cell proliferation. Gal-3 silencing in TPIN-SCs also diminished *in vitro* cell proliferation, thus substantiating a direct function of Gal-3 in supporting proliferation not only of differentiated cancer cells (18), but also of prostate CSCs, as previously described for other CSCs (23, 26). More importantly, Gal-3 impacted TPIN-SC proliferative potential also *in vivo*, as Gal-3 silencing in TPIN-SC reduced tumor burden. Thus, our findings confirm data obtained *in vitro* and *in vivo* with PC3 cells (18), and extend the role of Gal-3 to prostate CSCs. Gal-3 also supported the metastatic potential of TPIN-SCs, and when expressed in TPIN-SCs, Gal-3 endowed prostate CSCs with tropism for draining lymph nodes. Hence, when expressed in prostate CSCs, Gal-3 supports tumor growth and metastatic dissemination through cell-intrinsic and cell-extrinsic mechanisms (Figure 7). It will be interesting to identify the molecular mechanism by which Gal-3 endows CSCs with metastatic potential.

Gal-3 has been already implicated in the biology of prostate cancer (12), and Gal-3 has been proposed as predictive biomarker of prostate cancer aggressiveness especially in the context of metastasis (20, 21). Our analyses on human and mouse tissues confirm and extend previous findings showing that Gal-3 is expressed in both human and mouse PIN lesions, as well as in metastases (13–17, 63). All together, these findings suggest that Gal-3 has a relevant role already at the stage of PIN. Whereas, in humans a direct link between PIN and prostate adenocarcinoma has not been demonstrated, in TRAMP mice, PIN invariably precedes adenocarcinoma (31). Because Gal-3 is expressed in mouse PIN lesions, prostate CSCs and lymph node metastasis, we hypothesize that Gal-3 links mouse PIN lesions to lymph node metastasis *via* CSCs. By acting directly on CSCs and indirectly on immune surveillance, Gal-3 might favor the precocious dissemination of the former.

The finding that adenocarcinoma metastases are rare, but prostate CSCs can frequently be isolated from prostate draining lymph nodes of TRAMP mice is only apparently contradicting. In fact, autochthonous tumors in TRAMP mice lack genetic alterations that drive full prostate cancer metastatization (64). Nonetheless, our findings in the TRAMP model support a process of early lymph node seeding by prostate CSCs, and let us hypothesize that in some prostate cancer patients, metastasis occurs very early, and may account for recurrence after radical prostatectomy (65). Thus, measuring Gal-3 expression in prostate biopsies and/or Gal-3 levels in blood might be an early predictor of metastatic disease. This hypothesis requires investigation in a prospective clinical trial.

Importantly, a more intense Gal-3 staining was found at the leading edge of lymph node metastasis in some human and mouse samples. These findings further support a relevant role for Gal-3 in the invading process, and let us hypothesize that the leading edge is rich in CSCs. Because flow cytometry and immunohistochemistry analyses showed that Gal-3 expression is not restricted to CSCs, an alternative hypothesis is that Gal-3 might be upregulated in more invading tumor cells irrespective of their differentiation status. We are currently investigating between these two non-mutually exclusive possibilities.

Gal-3 overexpression at the leading edge of the lymph node metastasis, where cancer cells and lymphocytes get directly in contact, might also exert an important immunomodulatory activity. In support of this hypothesis, we have found that either silencing Gal-3 in TPIN-SC or the addition of LacNAc to the cultures rescued T cell proliferation in the presence of TPIN-SCs. More importantly, incubation of T cells with LacNAc rescued T cell proliferation in prostate-draining lymph nodes of TRAMP mice affected by mouse PIN. Thus, CSCs that early migrated to prostate-draining lymph nodes contributed to the establishment of an immunosuppressive milieu (30, 46) also through Gal-3. LacNAc in prostate-draining lymph nodes might inhibit Gal-3 either drained through lymphatic vessels from the primary tumor lesion, or locally produced by migrated prostate CSCs. Against the former hypothesis are evidences that inhibition of CSCs migration to prostate-draining lymph nodes avoids local immune suppression (30), and that CSCs require a cell-to-cell contact to dampen T cell activation (30).

Silencing of either Tenascin-C or Gal-3 in TPIN-SCs only partially rescued T cell proliferation, suggesting that the two molecules are endowed with different and potentially independent immunosuppressive mechanisms. In fact, Tenascin-C binds to α5β1 integrin expressed on the cell surface of both human and mouse T cells (30, 46), and inhibits stress fiber formation, thus dampening T cell receptor-dependent activation, proliferation, and cytokine production. Gal-3 has been reported to impair T cell activation by destabilizing the immunological synapse (66), and inhibiting TCR clustering (67) and CD8-TCR interactions (49). Our findings, however, do not exclude interactions between Gal-3 and Tenascin-C in favoring prostate CSCs tumorigenic and metastatic potential. As an example, by interacting with N-linked oligosaccharides on the surface of mammary carcinoma cells, Gal-3 induces activation of α5β1 integrin, the target of Tenascin-C on T cells (30), favoring, in this case, fibronectin fibrillogenesis, and fibronectin-dependent spreading and motility of tumor cells (68). Additionally, Gal-3 is able to directly interact with Tenascin-C (69), and the two molecules have been involved in homotypic cancer cell adhesion in glioma (70). Analogously, we speculate that Gal-3 and Tenascin-C may directly interact, thus mediating important steps of the metastatic cascade in prostate cancer.

The consistent number of Gal-3^+^ cells both in PIN lesions and in metastatic lymph nodes suggests that Gal-3 expression is not restricted to CSCs, and indeed, we have found that Gal-3 is also produced by more differentiated human and murine prostate cancer cells. Interestingly, intracellular distribution of Gal-3 appears to vary depending on the differentiation state of the cancer cells. While in less differentiated prostate CSCs, Gal-3 was equally distributed in nucleus and cytoplasm, in more differentiated cells Gal-3 was preferentially confined to the cytoplasm. In support of the latter finding, in most of the tumor cells in PIN and metastatic lesions, Gal-3 is cytoplasmic. The role of nuclear and cytoplasmic Gal-3 expression in prostate CSCs still need to be investigated. Within the cell, Gal-3 can shuttle between the nucleus and the cytoplasm, thus participating to cell cycle progression, cell growth, and apoptosis (18). We analyzed the apoptotic process in culture conditions, and found that TRAMP-C2 cells were more prone to apoptosis than CSCs, thus suggesting that nucleus-cytoplasm distribution of Gal-3 protects CSCs from apoptosis. This finding was unexpected, as cytoplasmic Gal-3 has been previously described to protect from apoptosis (71). Galectins can undergo post-translational proteolysis and phosphorylation (72), which might depend on the redox status of the microenvironment (73). Upon phosphorylation, Gal-3 acquires its anti-apoptotic and cell cycle arrest functions (74). Thus, we speculate that the culture conditions in which TRAMP-C2 cells were grown did not allow adequate post-translational modification of Gal-3. We also hypothesize that nuclear Gal-3 exclusion may associate with cell cycle progression in more differentiated prostate cancer cells. Thus, equal distribution of Gal-3 in the two cell compartments may favor CSC quiescence, and identify CSCs in prostate cancer. Further investigation is required to support our hypotheses.

Bresalier et al. (22) used surface Gal-3 expression as marker of CSCs in gastrointestinal tumors. Because we have found that both CSCs and more differentiated human and mouse prostate cancer cells express Gal-3 on the cell surface, surface Gal-3 does not appear to represent a stemness marker in prostate cancer.

Several Gal-3-specific therapeutic strategies are available (75). *In vitro*, PectaSol-C Modified Citrus Pectin and GCS-100 induced apoptosis, inhibition of cell proliferation and cell cycle arrest in cancer cells from the prostate and other tumors (76–78). Additionally, treatment with GCS-100 overcame the Gal-3-induced disfunction of tumor infiltrating T cells, and favored tumor rejection in mice (79). It has also been reported that the Thomsen-Friedenreich disaccharide TFD100 purified from cod blocked Gal-3-mediated angiogenesis and prostate cancer metastasis in mice, as well as apoptosis of activated T cells (80). Treatment with the Gal-3 inhibitor GR-MD-02 in combination with the stimulatory anti-OX40 monoclonal antibody promoted antigen specific T cell expansion and survival of mice bearing TRAMP-C1 tumors, reduced lung metastases in the 4T1 model, and showed anti-tumor activity in other mouse models (81). Gal-3 inhibitors, used either alone or in combination with immune checkpoint blockers or vaccination, have been and also are investigated in phase I-III clinical trials in melanoma, non-small cell lung cancer, squamous cell carcinoma of the head and neck, and chronic lymphocytic leukemia (NCT02575404, NCT02117362, NCT01723813, and NCT00514696). PectaSol-C Modified Citrus Pectin is proposed as dietary supplement in biochemical relapsed prostate cancer-affected patients [NCT01681823; refs. (75, 82)]. Our findings support the hypothesis that Gal-3 inhibitors also target CSCs, and could be tested in the early phases of prostate cancer.

## Data Availability Statement

The dataset has been uploaded to the GEO - GSE65502. Other raw data supporting the conclusions of this article will be made available by the authors, without undue reservation, to any qualified researcher.

## Ethics Statement

The studies involving human participants were reviewed and approved by Comitato Etico Ospedale San Raffaele. The patients/participants provided their written informed consent to participate in this study. The animal study was reviewed and approved by Institutional Ethical Committee, San Raffaele Hospital.

## Author Contributions

MB developed the concept of the study. MB, EJ, SC, ABre, AE, and CB designed and conceived the experiments. SC, MG, ABre, TB, AM, VP, AE, CB, and EJ performed the experiments. MG took care of the mouse colonies. AL supervised the experiments conducted to generate TPIN-SCkoGal-3 and TPIN-SCkoScram cell lines. IP analyzed microarray data. RG supervised western blot analyses. ABri provided patients samples. MF and CD supervised preparation of immunohistochemistry samples and analyzed them. SC and ABre prepared the figures and tables. MB and SC wrote and prepared the manuscript. All authors read and approved the manuscript.

## Conflict of Interest

The authors declare that the research was conducted in the absence of any commercial or financial relationships that could be construed as a potential conflict of interest.
