# Supplementary material for: Galectin-3 in Prostate Cancer Stem-Like Cells Is Immunosuppressive and Drives Early Metastasis
Source: Front Immunol. 2020 Sep 10;11:1820. doi: 10.3389/fimmu.2020.01820 (PMC7516304; doi:10.3389/fimmu.2020.01820)
Supplement: Supplementary file 1 [file Data_Sheet_1.docx]

Supplementary Material

Galectin-3 in prostate cancer stem-like cells is immunosuppressive and drives early metastasis

# Sara Caputo, Matteo Grioni, Chiara S. Brambillasca, Antonella Monno, Arianna Brevi, Massimo Freschi, Ignazio S. Piras, Angela R. Elia, Valentina Pieri, Tania Baccega, Angelo Lombardo, Rossella Galli, Alberto Briganti, Claudio Doglioni, Elena Jachetti, and Matteo Bellone

# Supplementary Figures and Tables

## Supplementary Figures

**
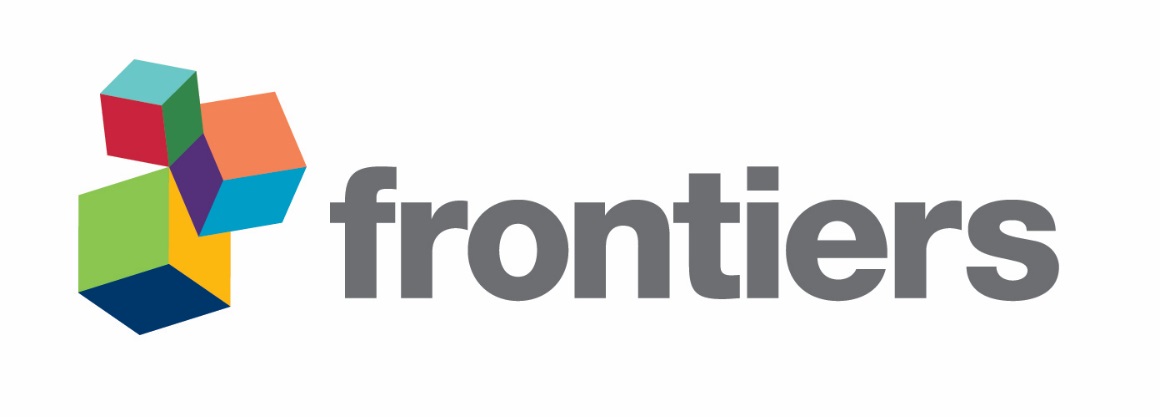
**

**Supplementary Figure 1. Gal-3 knockout in TPIN-SC. (**A) lgals3 knock out in TPIN-SC 1323 was induced by CRISPR/Cas9 technology. Panel (A) reports the scheme of the vector used to obtain Gal-3 knocking out (details are reported in material and methods). We obtained genetic knock out of Gal-3 in TPIN-SC. (B) Gal-3 expression in TPIN-SCkoGal-3 and TPIN-SCkoScram was assessed by flow cytometry analysis. Fresh samples for cell surface detection of Gal-3 were stained with anti-Gal-3 antibody and 7AAD. The plot reports representative histograms of Gal-3 staining, grey histogram: negative control for Gal-3 expression (TNE-SC). Panel (C) reports TPIN-SC 1323 phenotype. Fresh samples of TPIN-SC 1323 were stained with the indicated antibodies and 7AAD. The plots report representative histograms of stained sample (blue lines) and relative fluorescence minus one (FMO) sample (grey histograms).

**Supplementary Figure 2. Identification and isolation of CSCs and progenitor cells among TPIN-SC prostaspheres.** (A) TPIN-SCs were transduced with a lentiviral vector carrying the GFP sequence under the control of the Oct4 promoter. After the first cycle of FACS sorting, pure GFP^+^ TPIN-SCs in culture progressively gave rise to the original heterogeneous prostasphere composed of approximately 80% GFP^+^ and 20% GFP^-^ TPIN-SCs. After a second cycle of FACS sorting, GFP^+^ TPIN-SCs were isolated from the GFP^-^ TPIN-SCs counterparts. Expression of GFP and Oct4 in the two TPIN-SC populations was assessed by confocal microscopy, and representative images are reported in (B). Fixed and permeabilized samples were stained with Dapi, anti-Oct4 antibody and anti-GFP antibody, magnification 40X. Images were optimized for brightness/contrast using imageJ.

**Supplementary Figure 3. TPIN-SC are less prone to apoptosis than TRAMP-C2 cells.** TPIN-SC or TRAMP-C2 cells were plated at day 0 in standard culture conditions and apoptosis was analyzed at day 2 of culture by flow cytometry. Representative dot plots of Annexin V^+^7AAD^+^ cells. The results are representative of three independent experiments.

**Supplementary Figure 4. Gal-3 expression in human prostate cancer cell lines.** Surface expression of Gal-3 in the indicated human cells was assessed by FACS analysis. Fresh samples were stained with 7AAD. Cells were also stained with anti-Gal-3 antibody. Representative histograms of Gal-3 staining (blue lines); grey histograms: unstained.

**Supplementary Figure 5. TRAMP-C2 tumorigenic potential *in vivo*.** C57BL6/N mice were challenged subcutaneously with 2x10^6^ TRAMP-C2 (7 mice). The graph reports tumor growth (mm^3^) progression volume. Average ± SEM of tumor volume. Data are aggregate of two independent experiments.

**Supplementary Figure 6. Timeline of tumor growth (referring to Fig. 3 A).** Immunodeficient NSG mice received 2x10^6^ TPIN-SCshScram or TPIN-SCshGal-3#5 on day 0 (19 mice/group). Mice were constantly monitored for tumor growth. Fig. 3A reports the tumor dimension measured between day 35 and 44.

**Supplementary Figure 7. Gating strategy referred to Fig. 4 A.** Fresh samples for cell surface detection of Gal-3 were stained with anti-Gal-3 antibody and dead cells were excluded by 7AAD positivity. After gating on physical parameters to select single cells (FSC-A and FSC-H), 7AAD negative single cells were selected to analyze live cells only (FSC-A and 7AAD). Numbers reported on each plot illustrate the percentage of single cells (FSC-A and FSC-H) and the percentage of 7AAD negative single cells (FSC-A and 7AAD) for TPIN-SC and TRAMP-C2 cells respectively. Gal-3 staining shown in figure 4 A was obtained after gating in 7AAD negative cells. Representative dot plots of the gating strategy.


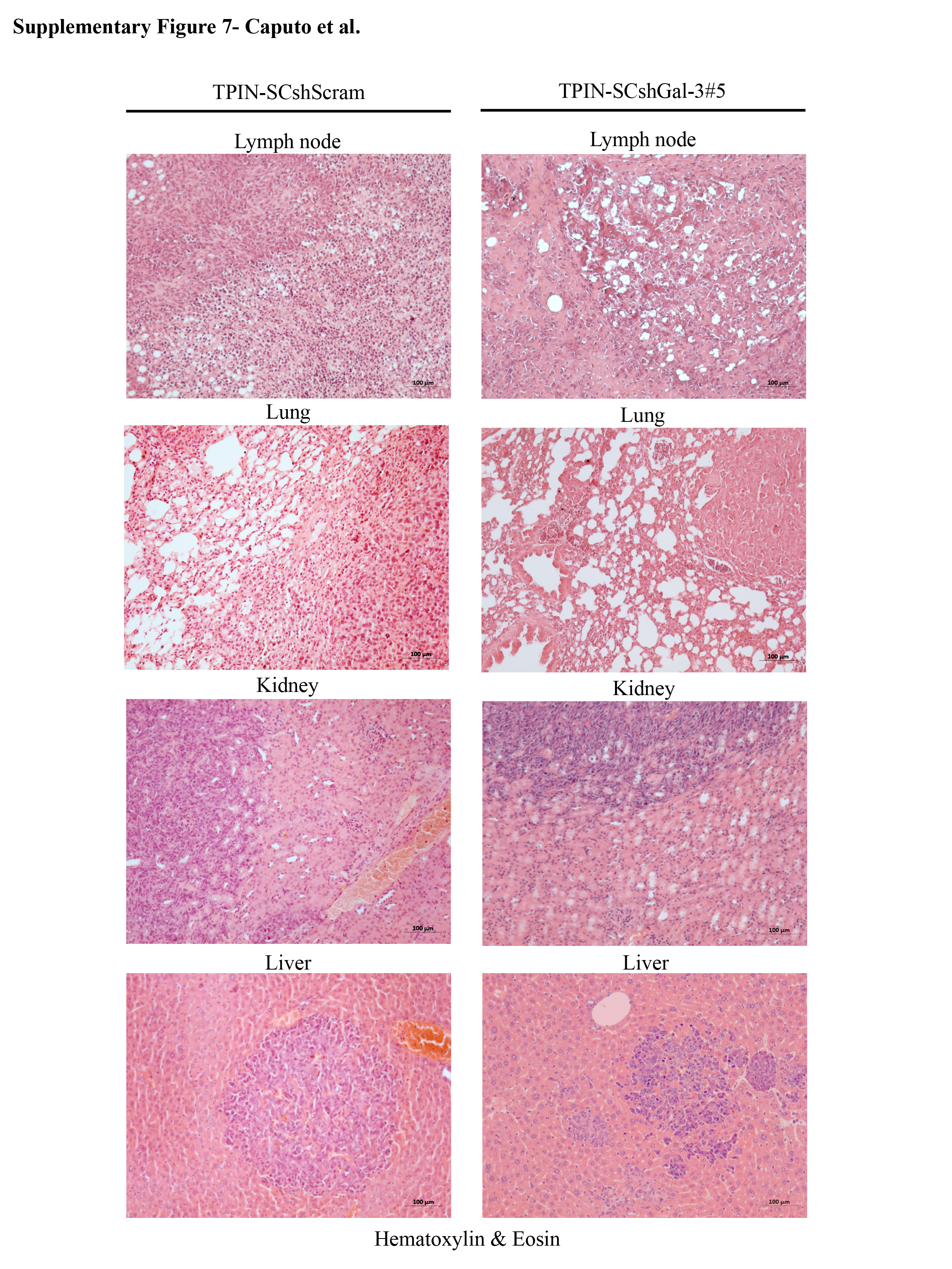


**Supplementary Figure 8. Metastatic organs from NSG mice injected with TPIN-SCshScram or TPIN-SCshGal-3#5.** Formalin fixed paraffin embedded sections from mice challenged with TPIN-SCshScram or TPIN-SCshGal-3#5. Metastatic lymph node, lung, kidney and liver were stained with hematoxylin and eosin and evaluated by an expert pathologist. Scale Bar =100 mm. Respectively for TPIN-SCshScram or TPIN-SCshGal-3#5 slides are representative of the following cases: Lymph node: 8 vs 4, Lung: 2 vs 1, Kidney: 2 vs 1, Liver: 2 vs 2. Images were optimized for brightness.
